# Significant improvement in catalytic activity and enantioselectivity of a *Phaseolus vulgaris* epoxide hydrolase, *Pv*EH3, towards *ortho*-cresyl glycidyl ether based on the semi-rational design

**DOI:** 10.1038/s41598-020-58693-1

**Published:** 2020-02-03

**Authors:** Chen Zhang, Youyi Liu, Chuang Li, Yaohui Xu, Yongjun Su, Jinping Li, Jun Zhao, Minchen Wu

**Affiliations:** 1 School of Pharmaceutical Science, Jiangnan University, Wuxi, 214122 China; 2 Wuxi School of Medicine, Jiangnan University, Wuxi, 214122 China; 3 School of Biotechnology, Jiangnan University, Wuxi, 214122 China; 4 The Affiliated Wuxi Matemity and Child Health Care Hospital of Nanjing Medical University, Wuxi, 214002 China

**Keywords:** Biological techniques, Biotechnology, Chemical biology, Microbiology

## Abstract

The investigation of substrate spectrum towards five racemic (*rac*-) aryl glycidyl ethers (**1a**–**5a**) indicated that *E. coli*/*pveh3*, an *E. coli* BL21(DE3) transformant harboring a *Pv*EH3-encoding gene *pveh3*, showed the highest EH activity and enantiomeric ratio (*E*) towards *rac*-**3a**. For efficiently catalyzing the kinetic resolution of *rac*-**3a**, the activity and *E* value of *Pv*EH3 were further improved by site-directed mutagenesis of selected residues. Based on the semi-rational design of an NC-loop in *Pv*EH3, four single-site variants of *pveh3* were amplified by PCR, and intracellularly expressed in *E. coli* BL21(DE3), respectively. *E. coli*/*pveh3*^E134K^ and /*pveh3*^T137P^ had the enhanced EH activities of 15.3 ± 0.4 and 16.1 ± 0.5 U/g wet cell as well as *E* values of 21.7 ± 1.0 and 21.2 ± 1.1 towards *rac*-**3a**. Subsequently, *E. coli*/*pveh3*^E134K/T137P^ harboring a double-site variant gene was also constructed, having the highest EH activity of 22.4 ± 0.6 U/g wet cell and *E* value of 24.1 ± 1.2. The specific activity of the purified *Pv*EH3^E134K/T137P^ (14.5 ± 0.5 U/mg protein) towards *rac*-**3a** and its catalytic efficiency (*k*_cat_/*K*_m_ of 5.67 mM^−1^ s^−1^) for (*S*)-**3a** were 1.7- and 3.54-fold those (8.4 ± 0.3 U/mg and 1.60 mM^−1^ s^−1^) of *Pv*EH3. The gram-scale kinetic resolution of *rac*-**3a** using whole wet cells of *E. coli*/*pveh3*^E134K/T137P^ was performed at 20 °C for 7.0 h, producing (*R*)-**3a** with 99.4% *ee*_s_ and 38.5 ± 1.2% yield. Additionally, the mechanism of *Pv*EH3^E134K/T137P^ with remarkably improved *E* value was analyzed by molecular docking simulation.

## Introduction

Enantiomeric isomers of chiral compounds, such as (*R*)- and (*S*)-enantiomers of a racemic drug, usually possess different and even antagonistic biological activities and pharmacological functions^1^. Since the early 1990s, there has been an ever-increasing demand for the optically pure epoxides and their corresponding vicinal diols, which are versatile and highly value-added building blocks applied diffusely in the pharmaceutical, fine chemical and agrochemical industries^2,3^. For examples, (*S*)-styrene oxide (SO) is a crucial drug intermediate for the synthesis of nematocide, anticancer agent — Levamisole, and anti-HIV agent — (−)-Hyperolactone C^4^, while (*R*)- and (*S*)-aryl/alkyl glycidyl ethers, such as (*S*)-benzyl glycidyl ether (**1a**) and (*R*)-*ortho*-cresyl glycidyl ether (**3a**), for the synthesis of chiral amino alcohols and β-blockers^5^.

Along with a green wave of global industrialization, the biocatalysis by whole cells or enzymes, having high enantio- and/or regio-selectivity and little or no byproducts, is recognized as an alternative or supplement to the chemocatalysis that requires hazardous metals and expensive chiral ligands, exampled by Jacobsen’s asymmetric ring-opening hydrolysis and epoxidation^6,7^. Epoxide hydrolases (EHs, EC 3.3.2.-), existing widely in organisms and being cofactor-independent catalysts, can enantio- and/or regio-selectively catalyze the opening of an active three-membered oxirane ring of racemic (*rac*-) epoxides, retaining single epoxide enantiomers and/or producing chiral vicinal diols^8^. The hydrolysis of epoxides by EHs mainly proceeds in two steps. A nucleophilic side-chain oxygen atom of Asp residue, one of the catalytic triad (Asp-His-Asp/Glu), in EH regioselectively attacks on the α- and β-carbon atoms (i.e., C_α_ and C_β_) in the oxirane ring of (*R*)- or (*S*)-epoxide, forming an EH−hydroxyalkyl intermediate. Then, a water molecule, activated by His residue located in EH’s catalytic triad, interacts with the intermediate, releasing a vicinal diol product^9^. Based on the catalytic mechanisms of the given EH−epoxide pairs, the asymmetric ring-opening hydrolysis of *rac*-epoxides was classified into two major pathways: both the kinetic resolution and enantioconvergent hydrolysis^10^.

The kinetic resolution of *rac*-epoxides by highly enantioselective EHs provides an environment-friendly bioprocess for the preparation of (*R*)- or (*S*)-epoxides with an intrinsic limitation of 50% yield^11^. However, the majority of wild-type (WT) EHs displayed unsatisfactory catalytic activity and/or *E* value, making them unable to be effectively used in the kinetic resolution of *rac*-epoxides^12^. With the development of protein engineering, the laboratory-directed evolution or modification of enzymatic structures based on the rational or semi-rational design has been applied to improving the catalytic characteristics of existing EHs and/or conferring the desired properties upon them^13^. Reportedly, EH222 with remarkably enhanced enantioselectivity, one mutant of a WT EH from *Aspergillus niger* (abbreviated to *An*EH), was obtained through four rounds of iterative saturation mutagenesis (ISM). The *E* value of EH222 towards *rac*-phenyl glycidyl ether (**2a**) was much higher than that of a WT *An*EH^14^. For another example, an EH from *Agrobacterium radiobacter* (*Ar*EH) was also subjected to ISM. Through high-throughput screening, the best mutant of *Ar*EH, T247K/I108L/D131S, was selected. Its catalytic efficiency (*k*_cat_/*K*_m_) and *E* value towards *rac*-epichlorohydrin were 4.5- and 2.1-fold higher than those of *Ar*EH^12^. Theoretically, the structural modification of EHs based on the rational or semi-rational design contributes to a better understanding on the molecular catalytic mechanism of the kinetic resolution of *rac*-epoxides.

**Figure 1:**
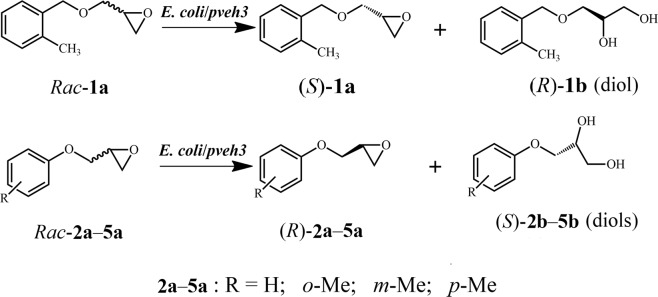
The substrate spectrum investigation of *E. coli*/*pveh3* towards *rac*-**1a**–**5a**: **1a**, benzyl glycidyl ether; **2a**, phenyl glycidyl ether; **3a**, *o*-cresyl glycidyl ether; **4a**, *m*-cresyl glycidyl ether; **5a**, *p*-cresyl glycidyl ether.

In our present work, the substrate spectrum investigation exhibited that *E. coli*/*pveh3*, an *E. coli* BL21(DE3) transformant intracellularly expressing *Pv*EH3 mediated by pET-28a(+), had the highest EH activity (6.03 ± 0.24 U/g wet cell) and *E* value (13.3 ± 0.7) towards *rac*-**3a** among all tested *rac*-**1a**–**5a** (Table 1 and Fig. 1). Through reviewing the experimental and analytical results reported previously^15,16^, the NC-loop, linking the N-terminal catalytic region with the cap domain, of *Pv*EH3 (GenBank accession no. ATG22745), was identified as the research object. To further improve the catalytic activity and *E* value of *Pv*EH3 towards *rac*-**3a**, four single- and one double-site variants of *pveh3* were amplified by two-stage whole-plasmid PCR^17^ based on the semi-rational design, and intracellularly expressed in *E. coli* BL21(DE3), respectively. Then, the EH activities and *E* values of all *E. coli* transformants as well as the specific activities of the purified *Pv*EH3, *Pv*EH3^E134K^, *Pv*EH3^T137P^ and *Pv*EH3^E134K/T137P^ towards *rac*-**3a** were determined, respectively. The kinetic parameters of *Pv*EH3^E134K/T137P^, such as Michaelis constant (*K*_m_) and turnover number (*k*_cat_), for (*S*)- and (*R*)-**3a** were also determined, and compared with those of a WT *Pv*EH3. To predict the reaction conditions for the kinetic resolution of *rac*-**3a**, the effects of pH and temperature on the activity and stability of *Pv*EH3^E134K/T137P^ were characterized. The preparative-scale production of (*R*)-**3a** via the kinetic resolution of *rac*-**3a** at high concentration was conducted using whole wet cells of *E. coli*/*pveh3*^E134K/T137P^. In addition, the molecular mechanism of *Pv*EH3^E134K/T137P^ with highly improved enantiopreference for (*S*)-**3a** was illuminated by analyzing and comparing the three-dimensional (3-D) structures of simulatedly docked EH–substrate complexes, such as *Pv*EH3− and *Pv*EH3^E134K/T137P^−(*S*)-**3a**.

**Table 1:** The substrate spectrum investigation of *E. coli*/*pveh3* towards *rac*-**1a–5a**.

Substrate	Activity (U/g wet cell)	Enantiopreference	*E* value	*ee*_s_ (%)	Yield (%)
*Rac*-**1a**	0.30 ± 0.01	(*R*)-**1a**	4.3 ± 0.2	99.0	18.2 ± 0.6
*Rac*-**2a**	4.59 ± 0.18	(*S*)-**2a**	10.4 ± 0.5	96.4	28.7 ± 0.8
*Rac*-**3a**	6.03 ± 0.24	(*S*)-**3a**	13.3 ± 0.7	98.4	31.2 ± 0.7
*Rac*-**4a**	3.83 ± 0.14	(*S*)-**4a**	8.2 ± 0.4	96.4	25.7 ± 0.9
*Rac*-**5a**	5.58 ± 0.23	(*S*)-**5a**	8.1 ± 0.4	97.0	25.6 ± 0.8

## Materials and Methods

### Plasmids, strains, and chemicals

*Escherichia coli* BL21(DE3) and plasmid pET-28a(+) (Novagen, Madison, WI) were applied to the construction of recombinant plasmids and expression of EH genes. Both a recombinant plasmid (pET-28a-*pveh3*) and a *Pv*EH3-expressing *E. coli* transformant (*E. coli*/*pveh3*) were constructed and preserved in our lab. PrimeSTAR HS DNA polymerase and *Dpn* I endonuclease (TaKaRa, Dalian, China) were applied to the single and double site-directed mutagenesis of *pveh3*. *Rac*-**1a**–**5a** were purchased from Energy (Shanghai, China), while (*S*)- and (*R*)-**3a** chemically synthesized by our lab according to the methods reported previously^18^. All other chemicals were of analytical grade, and commercially available from the local chemical companies (Wuxi, China).

### EH activity and protein assays

The activities of *Pv*EH3 and its mutants towards *rac*-**3a** were determined as described previously^19^, with slight modification. 950 μL cell suspension of 4.2 mg wet cells/mL or purified EH solution of 6.3 μg protein/mL, diluted with 100 mM Na_2_HPO_4_−NaH_2_PO_4_ buffer (pH 7.0), was well mixed with 50 μL 200 mM *rac*-**3a** dissolved in methanol (at a final concentration of 10 mM). The hydrolytic reaction was carried out at 20 °C for 10 min (for assaying the initial velocities of *Pv*EH3 and its mutants by controlling the conversion ratio of *rac*-**3a** within 10%) and terminated by the addition of 4 mL methanol. All the reaction samples were analyzed, respectively, by high-performance liquid chromatography (HPLC), using a Waters e2695 apparatus (Waters, Milford, MA) equipped with an XBridge BEH C18 column. Herein, the column temperature was set at 30 °C. The mobile phase of methanol/H_2_O (7:3, v/v) was used at 0.8 mL/min, and monitored by a Waters 2489 UV−Vis detector at 220 nm. One activity unit (U) of EH was defined as the amount of whole wet cells of *E. coli* transformant or purified EH hydrolyzing 1 μmol *rac*-**3a** per minute under the given assay conditions. Analogously, the EH activities of *E. coli*/*pveh3* were determined using 200 mg wet cells/mL towards 10 mM *rac*-**1a**, while 15 mg wet cells/mL towards 10 mM *rac*-**2a**, **4a** and **5a**, respectively.

SDS-PAGE was carried out according to the method of Laemmli^20^ on a 12.5% agarose gel, and the isolated proteins were visualized by staining with Coomassie Brilliant Blue R-250 (Sigma-Aldrich, St. Louis, MO). The apparent molecular weights of the expressed *Pv*EH3 and its mutants were estimated by comparison with those of standard proteins using a Quantity One software (https://www.bio-rad.com/). The enzyme protein concentration was determined with the BCA-200 protein assay kit (Pierce, Rockford, IL).

### Substrate spectrum assay of *Pv*EH3

The hydrolytic reactions of *rac*-epoxides, in aliquots of 2 mL 100 mM phosphate buffer (pH 7.0) system consisting of 20 mM *rac*-**1a–5a** and 30 mg wet cells/mL of *E. coli*/*pveh3*, were carried out at 20 °C, respectively. During the hydrolytic process, aliquots of 100 μL reaction sample were drawn out periodically, and extracted with 900 μL ethyl acetate. The extracted samples were analyzed by chiral HPLC equipped with a Chiralcel OD-H column (Daicel, Osaka, Japan) under the same operating conditions as stated above, except for using isopropanol/*n*-hexane (2:8, v/v) as the mobile phase. The absolute configurations of the single enantiomers of *rac*-**1a**−**2a** and **4a**−**5a** were confirmed, respectively, by comparing their retention times with those reported previously^10,18^. The conversion ratio (*c* value) of *rac*-substrate was defined as the percentage of its hydrolyzed amount to initial amount, while the yield of a single epoxide enantiomer, such as (*R*)-**3a**, was referred as the percentage of its retained amount to initial amount of *rac*-epoxide. The *ee*_s_ of retained single aryl glycidyl ether was calculated based on the equation: *ee*_s_ = [(*R* − *S*)/(*R* + *S*)] × 100%, where *R* and *S* represent the instantaneous concentrations of (*R*)- and (*S*)-**1a**–**5a**, respectively.

The enantioselectivity of EH towards a given *rac*-epoxide, being quantitatively described by its enantiomeric ratio (i.e., *E* value), was used to estimate the degree of enantiopreferential hydrolysis of one epoxide enantiomer over its antipode^21^. Based on the hydrolytic parameters of *rac*-epoxides (i.e., *c* and *ee*_s_ values) as defined and calculated above, *E* values of *E. coli*/*pveh3* towards *rac*-**1a**–**5a** (the substrate spectrum investigation), and those of the five *E. coli* transformants expressing *Pv*EH3’s mutants towards *rac*-**3a** (the screening of mutants) were calculated, respectively, using the equation: *E* = ln [(1 − *c*) × (1 − *ee*_s_)]/ln [(1 − *c*) × (1 + *ee*_s_)]^22^.

### Semi-rational design of the NC-loop in *Pv*EH3 for its site-directed mutagenesis

Using the known crystal structure of a *Solanum tuberosum* EH (*St*EH, PDB: 2CJP) at 1.95 Å resolution as a template, with which *Pv*EH3 shares 59.0% primary structure similarity, the 3-D structures of *Pv*EH3 and its best mutant, *Pv*EH3^E134K/T137P^, were homologically modeled using the MODELLER 9.21 program (https://salilab.org/modeller/)^23^, and then subjected to molecular mechanics optimization by CHARMM27 force field in the GROMACS 4.5 package (https://www.gromacs.org/)^24^. Meanwhile, the 3-D structures of substrates, (*S*)- and (*R*)-**3a**, were disposed in minimized energy using the ChemBioOffice 2010 package (https://www.cambridgesoft.com/)^25^.

The substrate spectrum investigation indicated that *E. coli*/*pveh3* showed the highest EH activity and *E* value towards *rac*-**3a** (Table 1), and preferentially hydrolyzed (*S*)-**3a** over its antipode. To further improve the catalytic activity and *E* value towards *rac*-**3a**, i.e., the enantiopreference for (*S*)-**3a**, *Pv*EH3 was subjected to site-directed mutagenesis of selected residues based on the semi-rational design. Firstly, the interaction between the modeled 3-D structures of *Pv*EH3 and (*S*)-**3a** was predicted by molecular docking (MD) simulation using the AutoDock vina program (https://autodock.scripps.edu/)^26^ to locate the most appropriate binding sites and steric orientation (having the lowest binding free energy). Then, the 3-D conformation of the docked enzyme−substrate complex, *Pv*EH3−(*S*)-**3a**, was optimized using the GROMACS 4.5 package, and visualized using a PyMol software (http://pymol.org/)^27^ to identify the amino acid residues in *Pv*EH3 in proximity to (*S*)-**3a** within 10 Å. Secondly, other four plant-derived EHs, *Vr*EH1, *Pv*EH1, *Pv*EH2 and *Nb*EH^3,28,29^, having superior catalytic activities and/or *E* values towards *rac*-**3a** and sharing over 65% identity with *Pv*EH3, were searched by a BLAST server in the Swiss-Prot Protein database (http://www.ebi.ac.uk/swissprot/). Then, the multiple sequence alignment among five plant EHs was performed using the Clustal Omega program (https://www.ebi.ac.uk/Tools/msa/clustalo/)^30^. Finally, considering the sites of non-conserved residues among five NC-loops, the several specific residues in an NC-loop of *Pv*EH3, in proximity to (*S*)-**3a** within 10 Å, were selected to be separately substituted with the corresponding and frequently emerging ones among other four EHs (identities equal to 75 and 100%).

### Construction of *E. coli* transformants harboring variant genes of *pveh3*

The site-directed mutagenesis of selected residues in *Pv*EH3’s NC-loop was carried out by two-stage whole-plasmid PCR. The PCR primers for the single and double site-directed mutagenesis of *pveh3* were designed according to *pveh3* sequence and the codons coding for mutation residues, and synthesized by Sangon (Shanghai, China) (Supplementary Table 1). Using a recombinant plasmid, pET-28a-*pveh*3, as the template, the first round of PCR was conducted with an upstream primer (such as E134K-U or T137P-U) and a downstream primer (pET-28a-D) as following conditions: an initial denaturation at 95 °C for 4 min, 30 cycles of at 98 °C for 10 s, 55 °C for 15 s and 72 °C for 3.5 min, and an extra elongation at 72 °C for 10 min. Then, the second round of PCR was continued using the first-round PCR product (a double-strand DNA) as megaprimers: 30 cycles of at 98 °C for 10 s, 55 °C for 15 s and 72 °C for 3 min. The amplified target PCR products, such as pET-28a-*pveh3*^E134K^ or -*pveh3*^T137P^, were digested with *Dpn* I endonuclease to decompose the methylated template, and then transformed into *E. coli* BL21(DE3), respectively. Thereafter, four resulting *E. coli* tranformants harboring single-site variants of *pveh3*, such as *E. coli*/*pveh3*^E134K^, were sent to Sangon company for DNA sequencing. Analogously, one recombinant plasmid connecting a double-site *pveh3* variant, pET-28a-*pveh3*^E134K/T137P^, was also amplified by PCR from pET-28a-*pveh3*^E134K^ using a pair of primers (T137P-U and pET-28a-D) and transformed into *E. coli* BL21(DE3), followed by DNA sequencing. One *E. coli* transformant containing a correct double-site variant gene was designated as *E. coli*/*pveh3*^E134K/T137P^.

### Expression of *Pv*EH3 and its mutants in *E. coli* BL21(DE3)

A single colony of *E. coli* transformant, such as *E. coli*/*pveh3*^E134K^, was inoculated into LB medium (1.0% tryptone, 0.5% yeast extract and 1.0% NaCl, pH 7.2) supplemented with 100 μg/mL kanamycin sulfate, and cultured at 37 °C overnight as the seed culture. Then, the same fresh medium was inoculated with 2% (v/v) seed culture, and cultured until OD_600_ value reached 0.6−0.8, followed by the addition of 0.05 mM IPTG to induce the expression of *Pv*EH3 or its mutant at 20 °C for 8 h. The *E. coli* transformant cells were collected by centrifugation (8000 rpm) at 4 °C, and resuspended in 100 mM Na_2_HPO_4_−NaH_2_PO_4_ buffer (pH 7.0) to 200 mg wet cells/mL used as the whole-cell biocatalyst unless stated otherwise. In this work, *E. coli*/*pveh3* was used as the positive control, while *E. coli* BL21(DE3) transformed with pET-28a(+), designated *E. coli*/pET-28a, as the negative control.

### Purification of the expressed *Pv*EH3 and its mutants

The collected *E. coli*/*pveh3*, /*pveh3*^E134K^, /*pveh3*^T137P^ or /*pveh3*^E134K/T137P^ cells, intracellularly expressing EH with a 6 × His tag at its N-terminus, were resuspended in buffer A (20 mM Tris–HCl, 500 mM NaCl and 20 mM imidazole, pH 7.5), and broken by ultrasonication in an ice-water bath. Then, the resulting supernatant was loaded onto a nickel–nitrilotriacetic acid (Ni-NTA) column (Tiandz, Beijing, China) preequilibrated with buffer A, followed by elution at a flow rate of 0.4 mL/min with buffer B as the same as buffer A except for 200 mM imidazole. Aliquots of 1.0 mL eluent only containing the target enzyme protein, *Pv*EH3, *Pv*EH3^E134K^, *Pv*EH3^T137P^ or *Pv*EH3^E134K/T137P^, analyzed by SDS-PAGE were pooled, dialyzed against 20 mM phosphate buffer (pH 7.0), and concentrated using a 10 kDa cut-off ultrafilter membrane (Millipore, Billerica, MA).

### Kinetic parameter assays of the purified *Pv*EH3 and *Pv*EH3^E134K/T137P^

The initial hydrolytic rates of (*S*)- and (*R*)-**3a** (μmol/min/mg protein) using 6.3 μg protein/mL purified *Pv*EH3 (or *Pv*EH3^E134K/T137P^) were determined, respectively, under the standard EH activity assay conditions, except for the concentrations of (*S*)- and (*R*)-**3a** ranging from 1.5 to 15 mM. The hydrolytic rate versus (*S*)- or (*R*)-**3a** concentration was plotted to verify whether the hydrolytic mode of *Pv*EH3 or *Pv*EH3^E134K/T137P^ conformed to Michaelis−Menten equation. Both the *K*_m_ and *V*_max_ values of *Pv*EH3 (or *Pv*EH3^E134K/T137P^) for (*S*)- and (*R*)-**3a** were calculated by non-linear regression analysis using an Origin 9.0 software (http://www.orignlab.com/). The *k*_cat_ value was deduced from the *V*_max_ and apparent molecular weight of EH, while the catalytic efficiency (*k*_cat_/*K*_m_) value was defined as the ratio of *k*_cat_ to *K*_m_. All the experimental data from three independent replicates were expressed as the mean ± standard deviation (SD).

### Effects of pH and temperature on the activity and stability of *Pv*EH3^E134K/T137P^

The pH optimum of the purified *Pv*EH3^E134K/T137P^ towards *rac*-**3a** was examined under the standard EH activity assay conditions, except for using different buffers (Na_2_HPO_4_−citric acid: pH 5.0–7.0 and Tris−HCl: pH 7.5–9.0). For estimating the pH stability, aliquots of *Pv*EH3^E134K/T137P^ solution were incubated at 20 °C for 1 h, in the absence of substrate, at a pH range of 5.0–9.0. The residual enzyme activity was determined under the standard EH activity assay conditions. Herein, the pH stability was defined as a pH range, over which the residual enzyme activity retained over 85% of its original activity.

The temperature optimum of *Pv*EH3^E134K/T137P^ towards *rac*-**3a** was measured, at pH optimum, at temperatures ranging from 15 to 45 °C. To evaluate the thermostability, aliquots of *Pv*EH3^E134K/T137P^ solution were incubated at the same temperature range for 1 h. The thermostability here was defined as a temperature, at or below which the residual enzyme activity was more than 85% of its original activity. Additionally, to ascertain a suitable reaction temperature for the kinetic resolution of *rac*-**3a**, the effect of temperature on the *E* value of *Pv*EH3^E134K/T137P^ was also investigated at 10–40 °C.

### Kinetic resolution of *rac*-3a using whole cells of *E. coli*/*pveh3*^E134K/T137P^

The gram-scale kinetic resolution of *rac*-**3a** at elevated concentrations was carried out using the whole wet cells of *E. coli*/*pveh3*^E134K/T137P^. The hydrolytic reactions, in aliquots of 20 mL 100 mM phosphate buffer (pH 7.0) system consisting of *rac*-**3a** at elevated concentrations ranging from 200 to 1000 mM and 200 mg wet cells/mL of *E. coli*/*pveh3*^E134K/T137P^, were conducted at 20 °C. During the hydrolytic process, aliquots of 50 μL reaction sample were periodically drawn out, extracted with 950 μL ethyl acetate, and analyzed by chiral HPLC for calculating the *c* value of *rac*-**3a** as well as the *ee*_s_, yield and space-time yield (STY) of retained (*R*)-**3a**. In this work, the STY (g/L/h) was defined as the amount of retained (*R*)-**3a** in per unit volume and time, and calculated based on the equation: STY = (M × C_0_ × Y)/*t*, in which C_0_ and *t* represent the initial concentration and hydrolytic time of *rac*-**3a**, respectively, while M and Y are the molar molecular weight and yield of retained (*R*)-**3a**.

### Molecular docking simulation of EH with (*S*)- or (*R*)-3a

The interaction between 3-D structures of *Pv*EH3 (or *Pv*EH3^E134K/T137P^) and (*S*)- or (*R*)-**3a** was predicted by MD simulation using the AutoDock vina program. On the basis of the 3-D conformations of the simulatedly docked enzyme−substrate complexes, such as *Pv*EH3− and *Pv*EH3^E134K/T137P^−(*S*)-**3a**, optimized using the GROMACS 4.5 package, the through-space distance (*d*_β_) and hydrogen bond length (*l*_1_ or *l*_2_) were identified by a PyMol software. Herein, the *d*_β_ was defined as the distance between a nucleophilic side-chain oxygen atom of Asp^101^ residue in *Pv*EH3 (or *Pv*EH3^E134K/T137P^) and a C_β_ (the less hindered terminal carbon atom in an oxirane ring) of (*S*)- or (*R*)-**3a**, while the *l*_1_ or *l*_2_ was the hydrogen bond length from a hydroxyl group of proton donor (Tyr^150^ or Tyr^232^) to an oxygen atom in the oxirane ring of epoxide. The binding free energy of *Pv*EH3 (or *Pv*EH3^E134K/T137P^) simulatedly docking with (*S*)- or (*R*)-**3a**, which is closely related to the affinity between them, was calculated using the molecular mechanics Poisson−Boltzmann surface area (MM-PBSA) method^22^.

## Results and discussion

### Catalytic activities and *E* values of *Pv*EH3 towards *rac*-1a–5a

In our previous studies, *pveh3* was cloned and expressed. To efficiently conduct the enantioconvergent hydrolysis, the catalytic activity of *Pv*EH3 towards *rac*-*para*-chlorostyrene oxide (*p*CSO) and its regioselectivity for (*S*)-*p*CSO were improved by site-saturation and mutiple site-directed mutagenesis^31,32^. The regioselectivities, quantitatively described by selectivity coefficients, β_*R*_ and α_*S*_, were adopted to evaluate the probabilities attacking on the C_β_ of (*R*)-epoxide and C_α_ of (*S*)-epoxide, respectively. It should be stressed that one specific EH is not likely to optimally function on all *rac*-epoxides and/or on two kinds of hydrolytic pathways towards a given *rac*-substrate. In this work, to expand the industrial application of *Pv*EH3 in the kinetic resolution of *rac*-aryl glycidyl ethers, the hydrolytic processes of *rac*-**1a**–**5a** using whole wet cells of *E. coli*/*pveh3* (Supplementary Figs. 1–5) and the substrate spectrum of *Pv*EH3 towards *rac*-**1a**–**5a** were investigated. As shown in Table 1, *E. coli*/*pveh3* enantiopreferentially catalyzed the hydrolysis of (*R*)-**1a** but (*S*)-**2a**–**5a**, and possessed the highest EH activity of 6.03 ± 0.24 U/g wet cell and *E* value of 13.3 ± 0.7 towards *rac*-**3a**, indicating that different aryl groups in aryl glycidyl ethers brought remarkable effects on the catalytic activity and *E* value of *Pv*EH3. Unfortunately, the catalytic properties, especially the *E* value, of *Pv*EH3 were still  unsatisfactory even towards *rac*-**3a**. Therefore, it is highly desirable to further improve its activity and *E* value by site-directed mutagenesis of selected residues for efficiently producing (*R*)-**3a** via the kinetic resolution of *rac*-**3a**. To the best of our knowledge, several EHs have been applied to the kinetic resolution of *rac*-aryl/alkyl glycidyl ethers, but they exhibited unsatisfactory catalytic properties towards *rac*-**3a**. For examples, the EH activity of *E. coli*/*vreh3*, an *E. coli* transformant harboring a *Vr*EH3-encoding gene *vreh3*, was only 4.8 U/g wet cell, while no activity of a *Tsukamurella paurometabola* EH (*Tp*EH1) was detected towards *rac*-**3a**^22,33^.

### Selection of the specific residues in the NC-loop of *Pv*EH3

**Figure 2:**
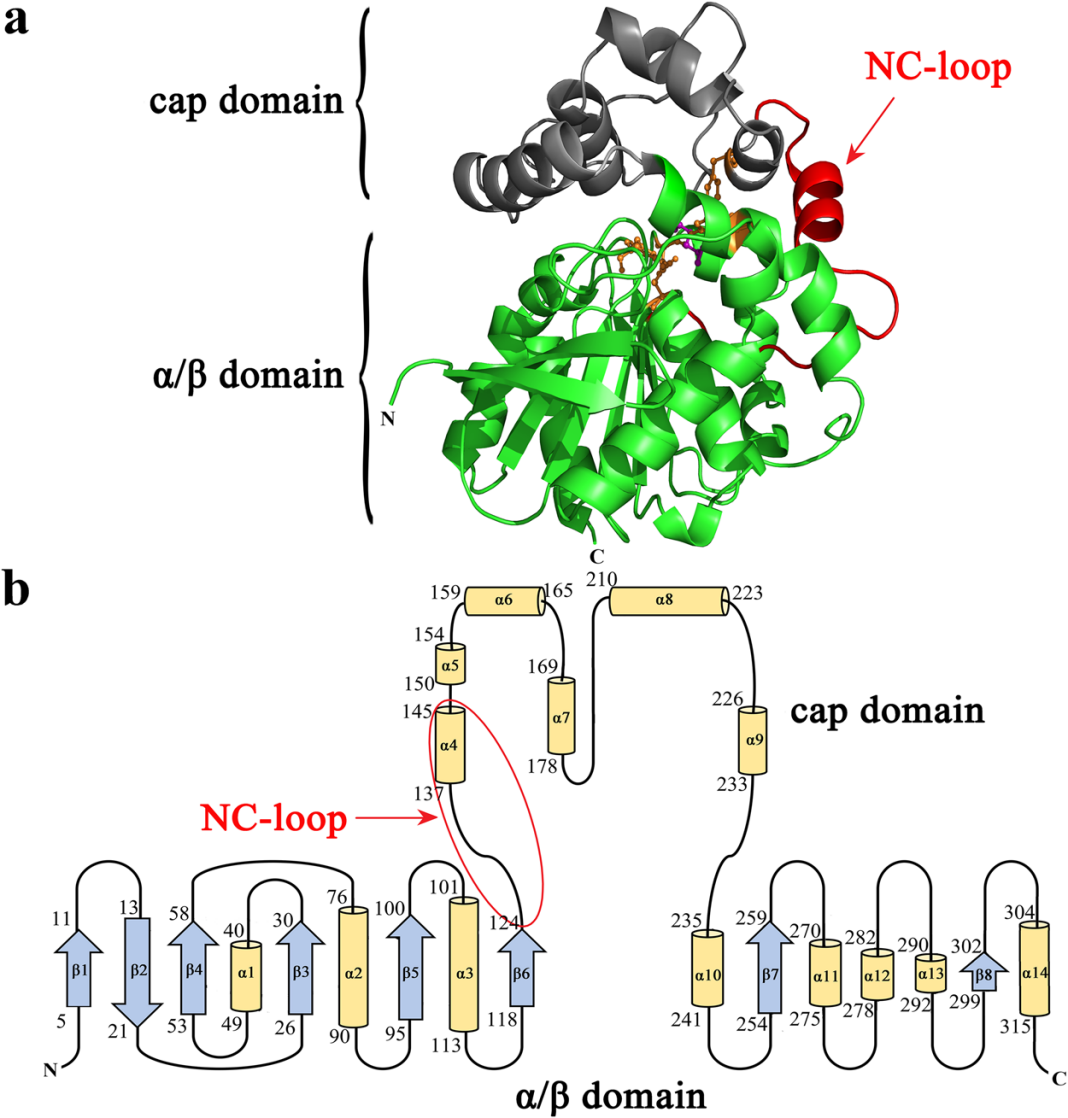
The molecular structure of *Pv*EH3. (**a**) The 3-D structure of *Pv*EH3 was homologically modeled by MODELLER 9.21 program based on the known crystal structure of *St*EH (PDB: 2CJP). Both the α/β and cap domains were indicated in green and gray, respectively, while the NC-loop in red. One catalytic triad and two proton donors were shown by sticks. (**b**) The topology diagram of *Pv*EH3’s secondary structures was deduced from its 3-D structure. The NC-loop was circled by red line.

The majority of characterized EHs, belonging to an α/β-hydrolase fold superfamily, were structurally divided into two main regions: an α/β domain, i.e., a β-sheet surrounded by a cluster of α-helices, and a cap domain. Both α/β and cap domains are connected by a variable NC-loop in amino acid composition and peptide chain length. Some experimental and analytical results have confirmed that the NC-loops of EHs are closely related to their catalytic characteristics^34,35^. For example, the laboratory-directed evolution study on the *A. niger* M200 EH demonstrated that Leu^215^ and Ala^217^, which are located in the NC-loop, remarkably affected its catalytic activity and *E* value towards *rac*-SO^15^. For another example, S-B17C*10 (a multiple-site mutant of *St*EH), whose *E* value towards *trans*-2-methylstyrene oxide was 1.93-fold than that of S-B17 (an *St*EH mutant), was obtained through two rounds of ISM. The structural analysis indicated that the mutation site, Leu^145^, in the NC-loop might remarkably influence the enantioselectivity (i.e., *E* value) of *St*EH^16^. In view of the above reported results, the NC-loop of *Pv*EH3 in this work was identified as the research object. Therefore, the 3-D structure of *Pv*EH3 was homologically modeled using the MODELLER 9.21 program (Fig. 2a), from which the topology diagram of secondary structures was deduced (Fig. 2b). As shown in Supplementary Fig. 6, *Pv*EH3 contains an NC-loop of 24 amino acid residues located at the sites from Ser^125^ to Asp^148^, which harbors a short α-helix.

**Figure 3:**
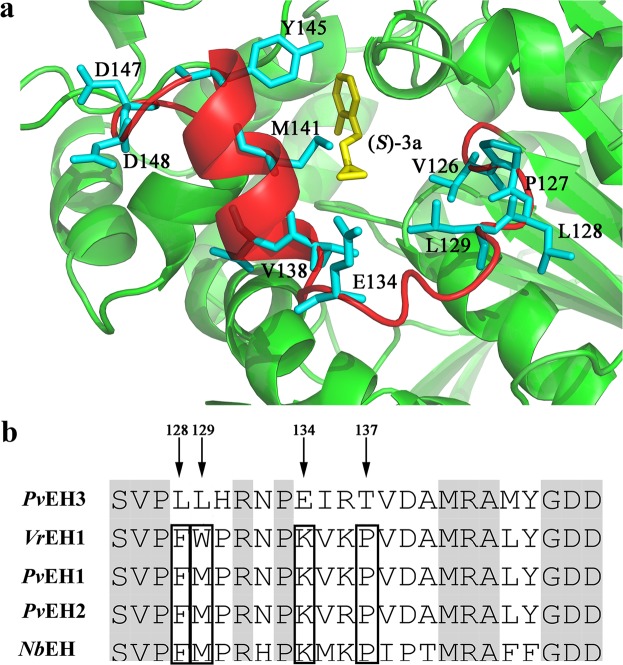
The selection of the specific residues in the NC-loop of *Pv*EH3. (**a**) The locally magnified 3-D conformation of *Pv*EH3−(*S*)-**3a**. Among 87 residues in *Pv*EH3 in proximity to (*S*)-**3a** within 10 Å, 11 residues (shown by blue sticks) were located in the NC-loop. (**b**) The multiple sequence alignment among NC-loops of five plant EHs. Four specific residues, L128, L129, E134 and T137, of *Pv*EH3 were indicated by arrows, while the corresponding and frequently emerging ones, F, M, K and P, among other four EHs (the identity equal to 75 or 100%) were boxed.

A total of 87 residues in *Pv*EH3 in proximity to (*S*)-**3a** within 10 Å were identified using a PyMol software based on the 3-D comformation of a simulatedly docked complex *Pv*EH3−(*S*)-**3a**. Among them, 11 residues were found to be located in the *Pv*EH3’s NC-loop (Fig. 3a). Furthermore, according to the result of multiple sequence alignment of the NC-loop of *Pv*EH3 with those of four selected plant EHs (Fig. 3b), five absolutely conserved residues in the NC-loops (Val^126^, Pro^127^, Met^141^, Asp^147^ and Asp^148^, sharing 100% identity with the corresponding residues in other four EHs) were eliminated from consideration. After further considering the remaining six non-conserved residues, two residues were also excluded from consideration because the two highest identity residues, Val^138^ and Tyr^145^, are the same as those of *Pv*EH3. Consequently, four specific residues, Leu^128^, Leu^129^, Glu^134^ and Thr^137^, in the NC-loop of *Pv*EH3 were selected to be separately substituted with the corresponding and frequently emerging residues, Phe, Met, Lys and Pro, among other four plant EHs (identities equal to 75 and 100%).

### Construction and screening of *E. coli* transformants harboring variants

Based on the above semi-rational design, four recombinant expression plasmids harboring single-site variant genes, such as pET-28a-*pveh3*^L128F^, were amplified by whole-plasmid PCR, and then transformed into *E. coli* BL21(DE3), respectively, resulting in the corresponding *E. coli* transformants, designated *E. coli*/*pveh3*^L128F^, /*pveh3*^L129M^, /*pveh3*^E134K^ and /*pveh3*^T137P^. SDS-PAGE analysis showed that *Pv*EH3 and its four mutants were successfully expressed, respectively, while no target band was detected in *E. coli*/pET-28a (Supplementary Fig. 7). Furthermore, the EH activities and *E* values of these transformants towards *rac*-**3a** were determined, respectively. Both *E. coli*/*pveh3*^E134K^ and /*pveh3*^T137P^ showed the improved EH activities of 15.3 ± 0.4 and 16.1 ± 0.5 U/g wet cell as well as *E* values of 21.7 ± 1.0 and 21.2 ± 1.1. Comparatively, both the EH activity and *E* value of *E. coli*/*pveh3* were 6.0 ± 0.2 U/g wet cell and 13.3 ± 0.7, respectively, under the same expression conditions (Table 2).

**Table 2:** The EH activities and *E* values of six *E. coli* transformants towards *rac***-3a**.

*E. coli* transformant	Activity (U/g wet cell)	*E* value
*E. coli*/*pveh3*	6.0 ± 0.2	13.3 ± 0.7
/*pveh3*^L128F^	7.1 ± 0.3	15.5 ± 0.8
/*pveh3*^L129M^	7.0 ± 0.2	10.8 ± 0.5
/*pveh3*^E134K^	15.3 ± 0.4	21.7 ± 1.0
/*pveh3*^T137P^	16.1 ± 0.5	21.2 ± 1.1
/*pveh3*^E134K/T137P^	22.4 ± 0.6	24.1 ± 1.2

Reportedly, there was a cooperative impact on the improvement in catalytic activity and *E* value of EHs via combinatorial or multiple-site mutagenesis^36,37^. Hence, to further improve the catalytic characteristics of *Pv*EH3 for the kinetic resolution of *rac*-**3a** at the elevated concentration, it was subjected to combinatorial substitution of both E134K and T137P based on the results of single site-directed mutagenesis. The recombinant plasmid, pET-28a-*pveh3*^E134K/T137P^, connecting a double-site variant *pveh3* was also amplified by whole-plasmid PCR as designed theoretically, transformed into *E. coli* BL21(DE3), and expressed successfully. As expected, *E. coli*/*pveh3*^E134K/T137P^ had the highest EH activity of 22.4 ± 0.6 U/g wet cell and *E* value of 24.1 ± 1.2, which were 3.7- and 1.8-fold those of *E. coli*/*pveh3* (Table 2). The EH activity determination along with SDS-PAGE analysis indicated that, compared with that of *E. coli*/*pveh3*, the remarkably improved EH activities of *E. coli*/*pveh3*^T137P^ and /*pveh3*^E134K/T137P^ were partially attributed to the expression level increases of target proteins in them. It was also reported that the expression level of *Bacillus pumilus* laccase in *E. coli* BL21(DE3) was increased through the site-directed mutagenesis of it^38^.

### Specific activities and kinetic parameters of the purified *Pv*EH3 and its mutants

The expressed *Pv*EH3, *Pv*EH3^E134K^, *Pv*EH3^T137P^ and *Pv*EH3^E134K/T137P^ were purified to homogeneity, respectively, by one-step Ni-NTA affinity column chromatography, displaying single target protein bands on SDS-PAGE with the same apparent molecular weight of about 36.1 kDa (Supplementary Fig. 7), which was close to their respective theoretical ones (36,023, 36,022, 36,019 and 36,018 Da) predicted by ProtParam program (https://web.expasy.org/protparam/). The specific activities of the purified *Pv*EH3^E134K^, *Pv*EH3^T137P^ and *Pv*EH3^E134K/T137P^ towards *rac*-**3a** under the standard EH activity assay conditions were determined to be 12.8 ± 0.4, 10.6 ± 0.3 and 14.5 ± 0.5 U/mg protein, which were about 1.5-, 1.3- and 1.7-fold that (8.4 ± 0.3 U/mg protein) of a WT *Pv*EH3.

The kinetic parameters of the purified *Pv*EH3 (or *Pv*EH3^E134K/T137P^) for (*R*)- and (*S*)-**3a** were measured (Table 3). Both *Pv*EH3 and *Pv*EH3^E134K/T137P^ displayed lower *K*_m_^*S*^ values for (*S*)-**3a** than the corresponding *K*_m_^*R*^ values for (*R*)-**3a**, suggesting that two EHs enantiopreferentially hydrolyzed (*S*)-**3a**. Compared with those of *Pv*EH3, the *K*_m_^*S*^ and *K*_m_^*R*^ (3.29 ± 0.15 and 5.40 ± 0.21 mM) of *Pv*EH3^E134K/T137P^ decreased by 46.4 and 45.1%, while its *k*_cat_^*S*^ and *k*_cat_^*R*^ (18.67 ± 0.52 and 1.37 ± 0.06 s^−1^) increased by 90.1 and 22.3%. As a result, the catalytic efficieny (*k*_cat_^*S*^/*K*_m_^*S*^ = 5.67 mM^−1^ s^−1^) of *Pv*EH3^E134K/T137P^ was 3.54-fold that (1.60 mM^−1^ s^−1^) of *Pv*EH3 for (*S*)-**3a**. These changes in kinetic parameters suggested that the combinatorial substitution, E134K and T137P, in the NC-loop of *Pv*EH3 had a significantly positive effect on its enantiopreference for (*S*)-**3a**.

**Table 3:** The kinetic parameters of the purified *Pv*EH3 and *Pv*EH3^E134K/T137P^ for (*R*)- and (*S*)**-3a**.

Enzyme	(*R*)-3a			(*S*)-3a		
	*k*_cat_^*R*^ (s^−1^)	*K*_m_^*R*^ (mM)	*k*_cat_^*R*^/*K*_m_^*R*^ (mM^−1^ s^−1^)	*k*_cat_^*S*^ (s^−1^)	*K*_m_^*S*^ (mM)	*k*_cat_^*S*^/*K*_m_^*S*^ (mM^−1^ s^−1^)
*Pv*EH3	1.12 ± 0.05	9.84 ± 0.28	0.11	9.82 ± 0.26	6.14 ± 0.22	1.60
*Pv*EH3^E134K/T137P^	1.37 ± 0.06	5.40 ± 0.21	0.25	18.67 ± 0.52	3.29 ± 0.15	5.67

### pH and temperature characteristics of the purified *Pv*EH3^E134K/T137P^

**Figure 4:**
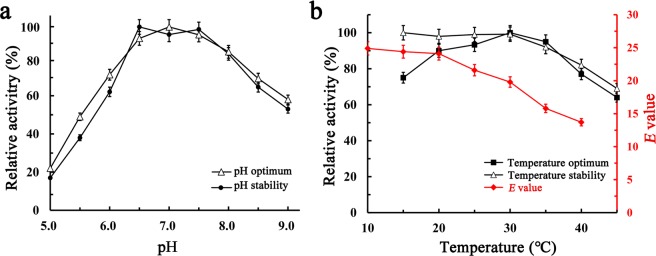
The pH and temperature characteristics of the purified *Pv*EH3^E134K/T137P^ towards *rac*-**3a**. (**a**) The pH optimum and stability of *Pv*EH3^E134K/T137P^ at a pH range of 5.0–9.0. (**b**) Its temperature optimum and stability at a range of 15 to 45 °C, while the effect of temperature on its *E* value at 10−40 °C.

*Pv*EH3^E134K/T137P^ displayed higher catalytic activity towards *rac*-**3a** at a pH range of 6.5–7.5, over which the highest catalytic activity (i.e., the pH optimum) was at pH 7.0. When the pH value was at 5.0 or 9.0, the catalytic activity of *Pv*EH3^E134K/T137P^ was only about 20 or 50% of that at pH 7.0. After incubation at 20 °C for 1 h at pH values of 5.0–9.0, *Pv*EH3^E134K/T137P^ exhibited higher stability (>85%) at a pH range of 6.5–7.5 (Fig. 4a).

The temperature optimum of *Pv*EH3^E134K/T137P^ towards *rac*-**3a**, at the pH optimum of 7.0, was determined to be 30 °C. After incubation at various temperatures (15–45 °C) for 1 h, *Pv*EH3^E134K/T137P^ retained over 85% of its original activity at 35 °C or below (Fig. 4b), which was similar to that of *A. mediolanus* EH^39^. Additionally, the effect of reaction temperature for the kinetic resolution on the *E* value of *Pv*EH3^E134K/T137P^ towards *rac*-**3a** was investigated at temperatures of 10–40 °C (Fig. 4b). The catalytic activity of *Pv*EH3^E134K/T137P^ was increased as the temperature was elevated up to 30 °C, while its *E* value was decreased all through as the temperature rising. As a result, a suitable temperature of 20 °C was selected for the kinetic resolution, at which the catalytic activity of *Pv*EH3^E134K/T137P^ was 85% of that at 30 °C while its *E* value was 24.1 ± 1.2. It is recognized that the lower the temperature, the higher the *E* value is. For examples, the *E* value of *A. usamii* EH towards *rac*-SO was elevated from 11.5 to 25.5 when the temperature was decreased from 35 to 0 °C, while the similar result was also observed in the case of the kinetic resolution of *rac*-SO by *Rhodotrorula glutinis* EH^40,41^.

### Preparative-scale production of (*R*)-3a by whole cells of *E. coli*/*pveh3*^E134K/T137P^

To realize the gram-scale preparation of (*R*)-**3a**, the enantioselective hydrolytic reactions of *rac*-**3a** at concentrations of 200, 400, 600, 800 and 1000 mM, in aliquots of 20 mL 100 mM phosphate buffer (pH 7.0) system, were investigated, respectively, at 20 °C using 200 mg wet cells/mL of *E. coli*/*pveh3*^E134K/T137P^. The reaction process was monitored by chiral HPLC at given intervals to confirm a suitable time for preventing the substrate from excessive hydrolysis. To our knowledge, using the whole cell instead of purified EH as the biocatalyst was because that the former was easily accessible and possessed higher stability^42^.

**Figure 5:**
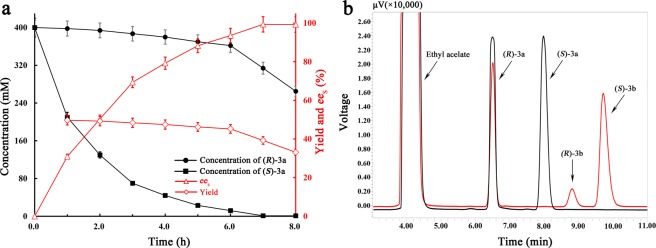
The gram-scale production of (*R*)-**3a** via the kinetic resolution of 800 mM *rac*-**3a** using 200 mg wet cells/mL of *E. coli*/*pveh3*^E134K/T137P^. (**a**) The progress curves of the kinetic resolution of *rac*-**3a** within 8.0 h. (**b**) The chiral HPLC traces of the initial *rac*-**3a** (black line) as well as the retained (*R*)-**3a** and produced diols (red line) after *rac*-**3a** was incubated at 20 °C for 7.0 h.

As shown in Table 4, *rac*-**3a** was efficiently and enantioselectively hydrolyzed until its concentration up to 800 mM, producing (*R*)-**3a** with *ee*_s_ over 99.0% and yield from 38.5 ± 1.2 to 40.2 ± 1.5% (a theoretical yield of 50%). However, when *rac*-**3a** concentration was raised to 1000 mM, the *ee*_s_ of (*R*)-**3a** was only 74.8 ± 2.1% until 14.0 h. The reason may be that there was a severe inhibition or denaturation effect on EHs when the substrate reached or exceeded a limit concentration^7,22^. Therefore, the maximum allowable concentration of *rac*-**3a** was 800 mM.  The hydrolytic process of 800 mM *rac*-**3a** was shown in Fig. 5. After *rac*-**3a** was incubated at 20 °C for 7.0 h at 61.4 ± 1.6% *c*, (*S*)-**3a** was almost completely hydrolyzed, while (*R*)-**3a** was retained with 99.4% *ee*_s_ and 38.5 ± 1.2% yield. The STY of (*R*)-**3a** was calculated to be 7.2 g/L/h, being 14.7- and 30.0-fold those by the whole cells of *Trichosporon loubierii* and *A. niger* expressing *Tl*EH and *An*EH^43,44^. As the hydrolytic reaction of *rac*-**3a** was continued until 8.0 h, the *ee*_s_ of (*R*)-**3a** had no obvious increase, but its yield and STY dropped to 33.2 ± 0.9% and 4.9 g/L/h, respectively. In conclusion, *rac*-**3a** at 800 mM was subjected to the kinetic resolution for 7.0 h, followed by purification using the silica gel column chromatography, obtaining (*R*)-**3a** with more than 99.0% *ee*_s_ and 32.6 ± 1.0% overall yield. The enantiopure (*R*)-**3a**: colorless oil; [α]_D_^20^: −13.56 (c 0.50, methanol), >99.0% *ee*_s_; ^1^H NMR (400 MHz, CDCl_3_, TMS): δ 7.13−7.16 (m, 2H), 6.9 (t, *J* = 7.6 Hz, 1H), 6.8 (d, *J* = 8.0 Hz, 1H), 4.25 (dd, *J*_1_ = 3.2 Hz, *J*_2_ = 11.2 Hz, 1H), 4.00 (q, *J* = 5.2 Hz, 1H), 3.36−3.40 (m, 1H), 2.92 (t, *J* = 4.4 Hz, 1H), 2.8 (dd, *J*_1_ = 2.8 Hz, *J*_2_ = 4.8 Hz, 1H), 2.25 (s, 3H).

**Table 4:** The gram-scale kinetic resolution of *rac*-**3a** using 200 mg wet cells/mL of *E. coli*/*pveh3*^E134K/T137P^.

*Rac*-3a conc. (mM)	Time (h)	*c* (%)	*ee*_s_ (%)	Yield (%)
200	1.8	59.7 ± 1.7	99.7	40.2 ± 1.3
400	4.0	59.9 ± 1.7	99.6	39.0 ± 1.2
600	5.0	60.8 ± 1.8	99.1	39.1 ± 1.1
800	7.0	61.4 ± 1.6	99.4	38.5 ± 1.2
1000	14.0	44.2 ± 1.3	74.8 ± 2.1	−

### Analysis of *Pv*EH3^E134K/T137P^ with obviously improved preference for (*S*)-3a

The substrate-binding pocket (SBP) of EHs, harboring a cluster of amino acid residues such as a catalytic triad and two proton donors, was located between the α/β and cap domains which are connected by an NC-loop^22^. It was reported that the spatial position and orientation of substrate-binding residues, influencing the catalytic activity and substrate preference, can be changed by specific residue substitutions in the NC-loop and even more distant regions^45,46^. In the case of *Pv*EH3 (or *Pv*EH3^E134K/T137P^), the substrate-binding residues are identified as one catalytic nucleophile (Asp^101^) and two proton donors (Tyr^150^ and Tyr^232^). The 3-D structure alignment between *Pv*EH3 and *Pv*EH3^E134K/T137P^ revealed that there were obvious distinctions in the steric position and orientation of Asp^101^, Tyr^150^ and Tyr^232^ (Supplementary Fig. 8), which may improved the affinity of *Pv*EH3^E134K/T137P^−(*S*)-**3a**.

**Figure 6:**
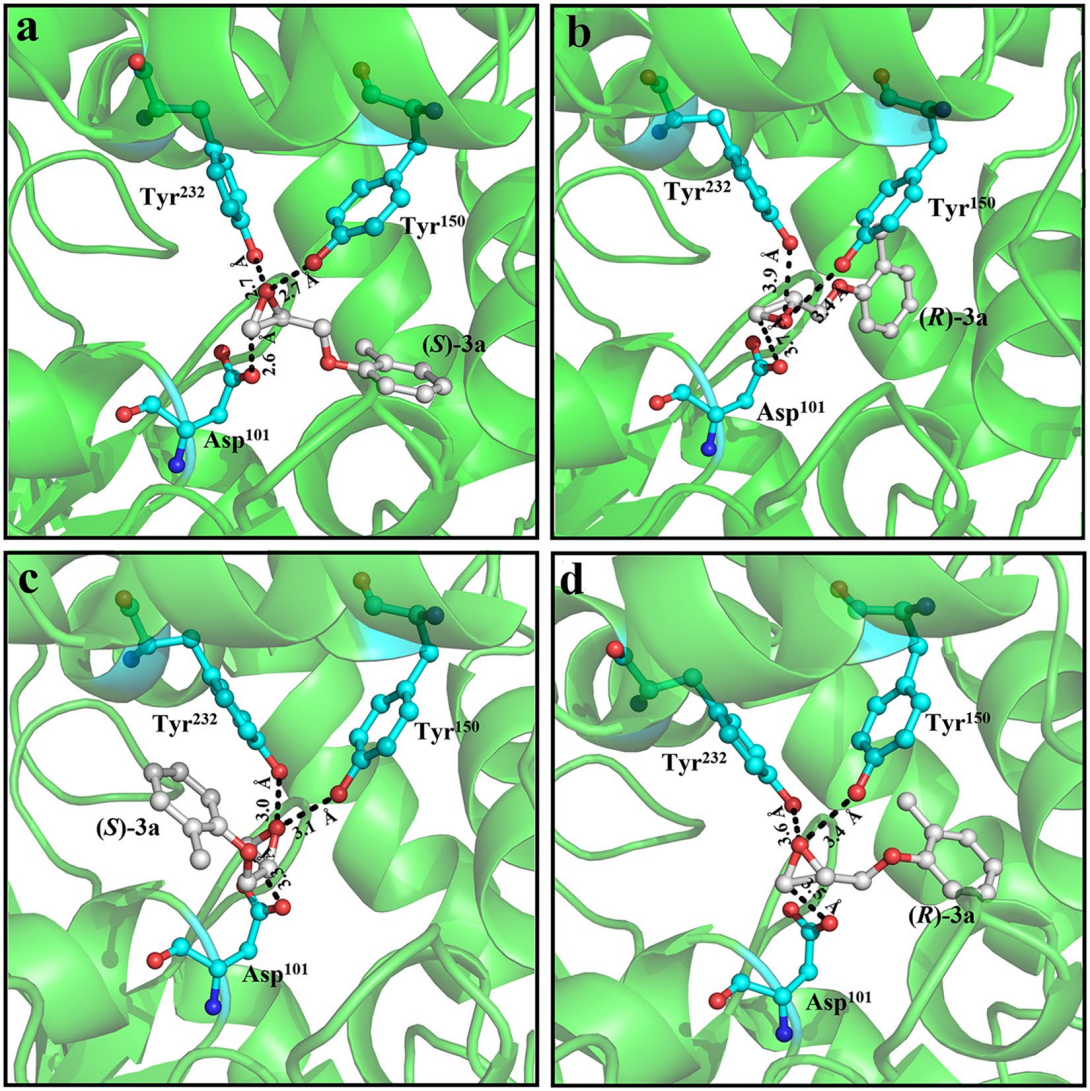
The molecular mechanism of *Pv*EH3^E134K/T137P^ with obviously improved enantiopreference for (*S*)-**3a**. The locally magnified 3-D conformations of *Pv*EH3^E134K/T137P^−(*S*)-**3a** (**a**) and −(*R*)-**3a** (**b**) were compared with those of *Pv*EH3−(*S*)-**3a** (**c**) and −(*R*)-**3a** (**d**), respectively.

The C_β_ of (*S*)- or (*R*)-**3a** was mainly attacked by side-chain oxygen atom of Asp^101^ residue, generating the corresponding (*S*)- or (*R*)-diol via the retention of configuration. Therefore, the *d*_β_ (the through-distance between oxygen atom and C_β_) was considered as a key parameter^47^. The 3-D conformations of *Pv*EH3^E134K/T137P^−(*S*)-**3a** and −(*R*)-**3a** were compared with those of *Pv*EH3−(*S*)-**3a** and −(*R*)-**3a**, respectively (Fig. 6). The *l*_1_ and *l*_2_ from the hydroxyl groups of Tyr^150^ and Tyr^232^ in *Pv*EH3 (or *Pv*EH3^E134K/T137P^) to the oxygen atom in the oxirane ring of (*S*)- or (*R*)-**3a** were less than 3.5 Å, which are the prerequisites for ring-opening hydrolysis^48^. The *d*_β_ value of *Pv*EH3− (or *Pv*EH3^E134K/T137P^−(*S*)-**3a**) was shorter than that of *Pv*EH3− (or *Pv*EH3^E134K/T137P^−(*R*)-**3a**), suggesting that *Pv*EH3 or *Pv*EH3^E134K/T137P^ preferentially catalyzes the hydrolysis of (*S*)-**3a**. Four docked enzyme−substrate complexes had approximate binding free energies from –18.23 to –17.85 kJ/mol predicted using AutoDock vina program, but the differences in *d*_β_ values were remarkable. The *d*_β_ value of docked *Pv*EH3^E134K/T137P^−(*S*)-**3a** was shortened to 2.6 Å from 3.3 Å of *Pv*EH3−(*S*)-**3a**, while that of *Pv*EH3^E134K/T137P^−(*R*)-**3a** was elevated to 3.7 Å from 3.5 Å of *Pv*EH3−(*R*)-**3a**. All the above explanations by analyzing and comparing the 3-D conformations of the simulatedly docked EH−substrate complexes were in accordance with our experimental measurements, and also similar to the conclusions drawn by other research groups^49,50^.

## Conclusions

Using an NC-loop in *Pv*EH3 as the research object, its single and double site-directed mutagenesis was carried out based on the semi-rational design. The specific residue substitution, E134K or T137P in the NC-loop, had a positive effect on the catalytic activity and *E* value of *Pv*EH3. Furthermore, *E. coli*/*pveh3*^E134K/T137P^ harboring a double-site variant of *pveh3* possessed the highest EH activity and *E* value towards *rac*-**3a** among all the tested *E. coli* transformants, suggesting that a combinatorial substitution, E134K and T137P, had a synergistic impact. Compared with *Pv*EH3, the purified *Pv*EH3^E134K/T137P^ displayed the obviously improved specific activity towards *rac*-**3a** and the enhanced catalytic efficiency (*k*_cat_^*S*^/*K*_m_^*S*^) for (*S*)-**3a**, The gram-scale kinetic resolution of 800 mM *rac*-**3a** was conducted using whole wet cells of *E. coli*/*pveh3*^E134K/T137P^, producing (*R*)-**3a** with high *ee*_s_, yield and STY. Furthermore, the molecular mechanism of *Pv*EH3^E134K/T137P^ with markedly improved enantiopreference for (*S*)-**3a** was explained by MD simulation. Our present work provided an efficient technical strategy to customize the desired EHs for preparing target chiral epoxides.

## Supplementary information

Supplementary information.
