# Supplementary material for: Significant improvement in catalytic activity and enantioselectivity of a Phaseolus vulgaris epoxide hydrolase, PvEH3, towards ortho-cresyl glycidyl ether based on the semi-rational design
Source: Sci Rep. 2020 Feb 3;10:1680. doi: 10.1038/s41598-020-58693-1 (PMC6997370; doi:10.1038/s41598-020-58693-1)
Supplement: Supplementary file 1 — Supplementary information. [file 41598_2020_58693_MOESM1_ESM.docx]

**Significant improvement in catalytic activity and enantioselectivity of a** ***Phaseolus vulgaris* epoxide hydrolase, *Pv*EH3, towards *ortho*-cresyl glycidyl ether based on the** **semi-rational design**

Chen Zhang^1^, Youyi Liu^2^, Chuang Li^3^, Yaohui Xu^4^, Yongjun Su^1^, Jinping Li^2^, Jun Zhao^4^ & Minchen Wu^2^

^1^ School of Pharmaceutical Science, Jiangnan University, Wuxi, 214122, China.

^2^ Wuxi School of Medicine, Jiangnan University, Wuxi, 214122, China.

^3^ School of Biotechnology, Jiangnan University, Wuxi, 214122, China.

^4^ The Affiliated Wuxi Matemity and Child Health Care Hospital of Nanjing Medical University, Wuxi, 214002, China.

Chen Zhang and Youyi Liu, the two first authors, contributed equally to this work.

Correspondence and requests for materials should be addressed to M.W. (email: [biowmc@126.com](mailto:biowmc@126.com)) or J.Z. (email: [chalange@163.com](mailto:chalange@163.com))

**Contents**

**Supplementary Table 13**

**Supplementary Fig. 14**

**Supplementary Fig. 24**

**Supplementary Fig. 35**

**Supplementary Fig. 45**

**Supplementary Fig. 56**

**Supplementary Fig. 67**

**Supplementary Fig. 78**

**Supplementary Fig. 88**

**Supplementary Data**

**Supplementary Table 1.**

**PCR primers for the single and double site-directed mutagenesis of *pveh3*.**

| Primer name | Primer sequence (5′→3′) ^a^ |
| --- | --- |
| L128F-U | CTCAGTGTGCCTTTCCTCCACCGAAAC |
| L129M-U | AGTGTGCCTATGCICCACCGAAACCCC |
| E134K-U | CTCCACEGTAACCCCAAGATCAGAACCG |
| T137P-U | AACCCOGAGATCAGACCAGTCGATGCCA |
| pET-28a-D | GCCTTACTGGTTAGCAGAATG |

^a^ The codons encoding mutation residues are boxed.


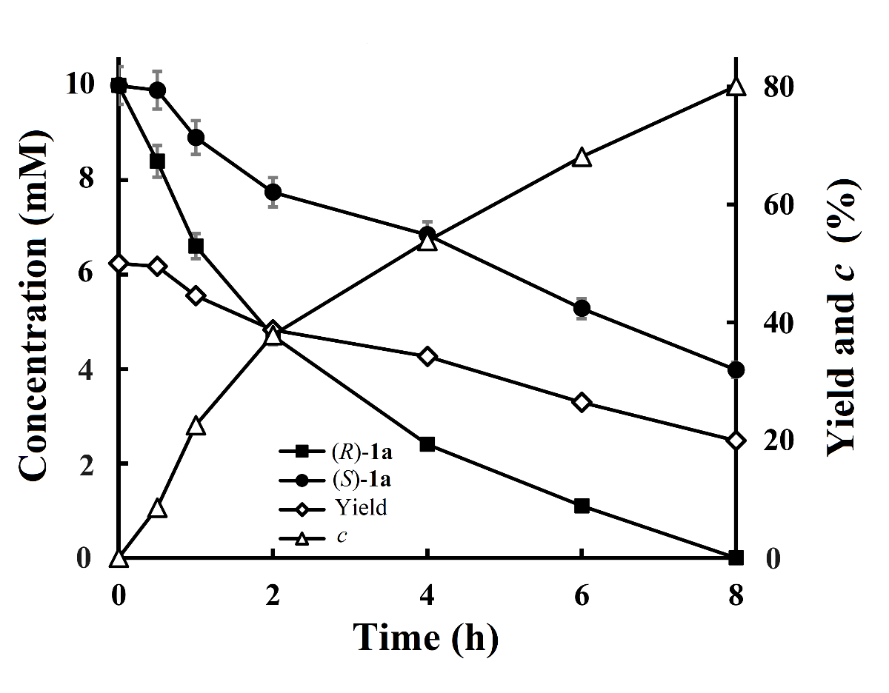


**Supplementary Fig. 1. The progress curves of the kinetic resolution of 20 mM *rac*-1a using 80 mg wet cells/mL of *E. coli*/*pveh3*.**


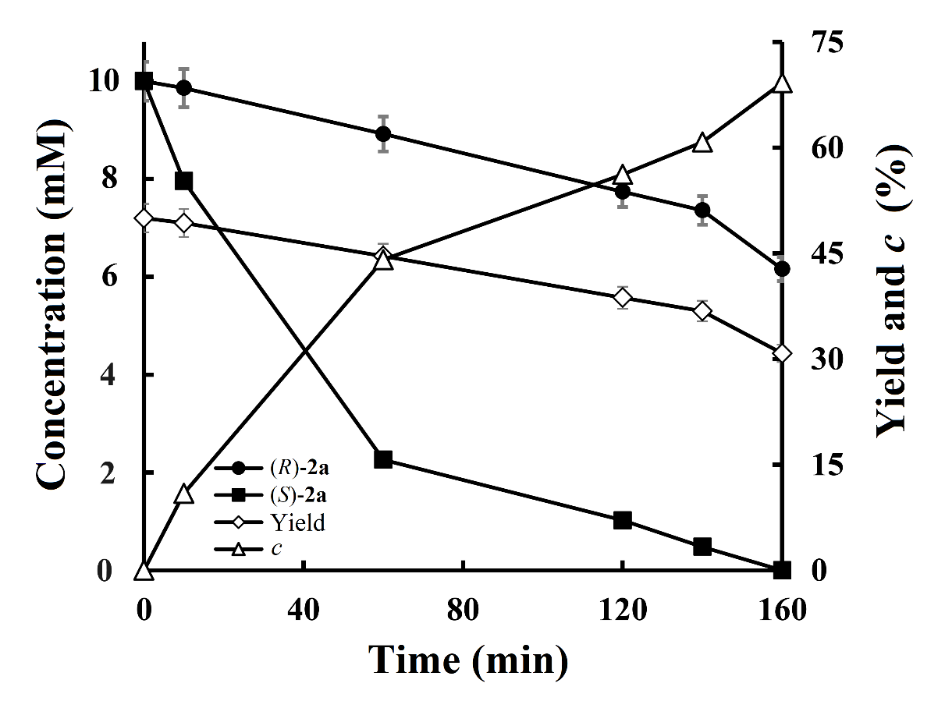


**Supplementary Fig. 2. The progress curves of the kinetic resolution of 20 mM *rac*-2a** **using 80 mg wet cells/mL of *E. coli*/*pveh3*.**


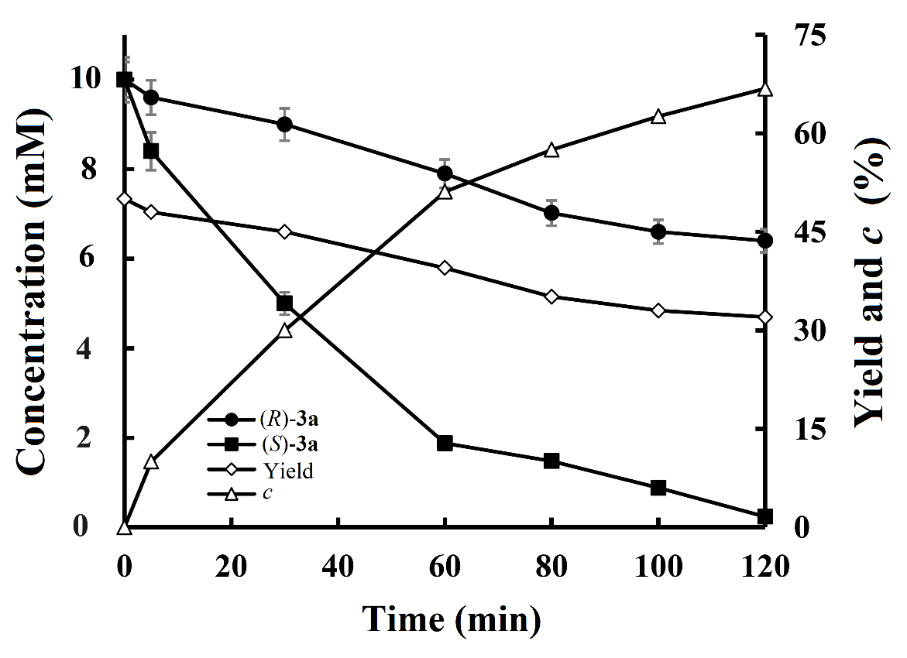


**Supplementary Fig. 3. The progress curves of the kinetic resolution of 20 mM *rac*-3a using 80 mg wet cells/mL of *E. coli*/*pveh3*.**


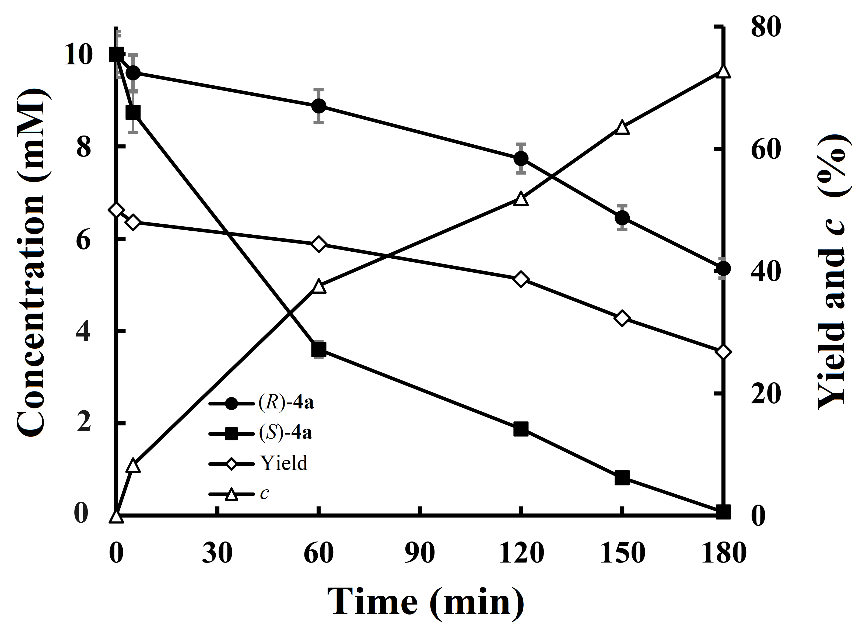


**Supplementary Fig. 4. The progress curves of the kinetic resolution of 20 mM *rac*-4a using 80 mg wet cells/mL of *E. coli*/*pveh3*.**


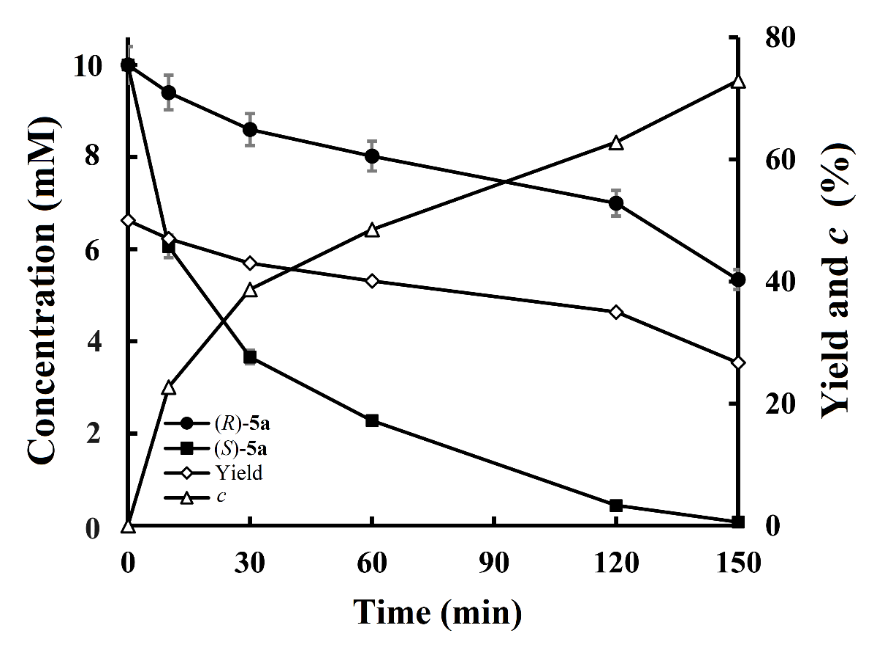


**Supplementary Fig. 5. The progress curves of the kinetic resolution of 20 mM *rac*-5a using 80 mg wet cells/mL of *E. coli*/*pveh3*.**

**
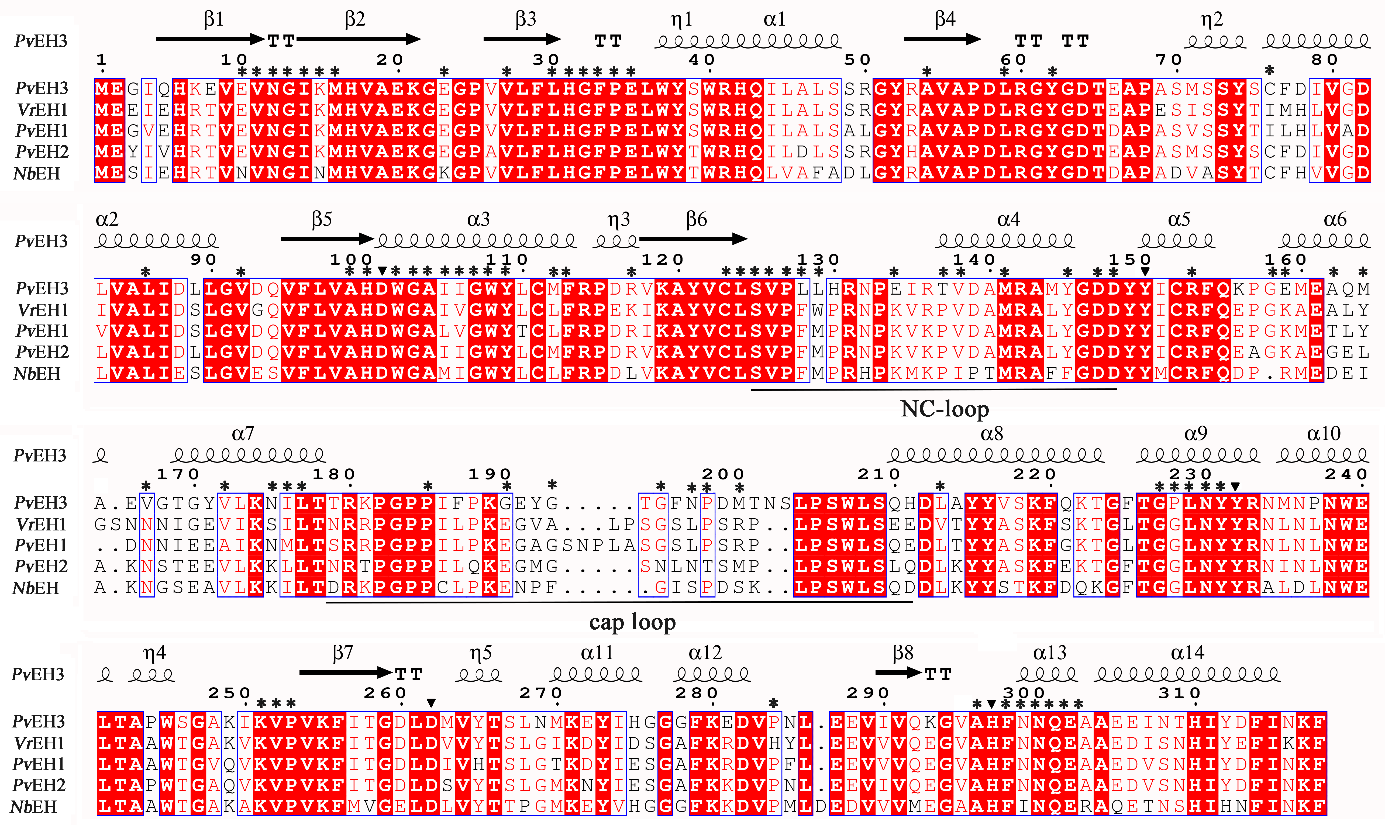
**

**Supplementary Fig. 6. The multiple sequence alignment among five plant EHs.** *Pv*EH3 in this work, ATG22745; *Vr*EH1, ADP68585; *Pv*EH1, AKJ75509; *Pv*EH2, ASS33914 and *Nb*EH, ACE82566. One catalytic triad (Asp^101^ -His^297^-Asp^262^) and two proton donors (Tyr^150^ and Tyr^232^) in *Pv*EH3 were marked by inverted triangles, while other 82 residues in proximity to (*S*)-**3a** within 10 Å by stars.


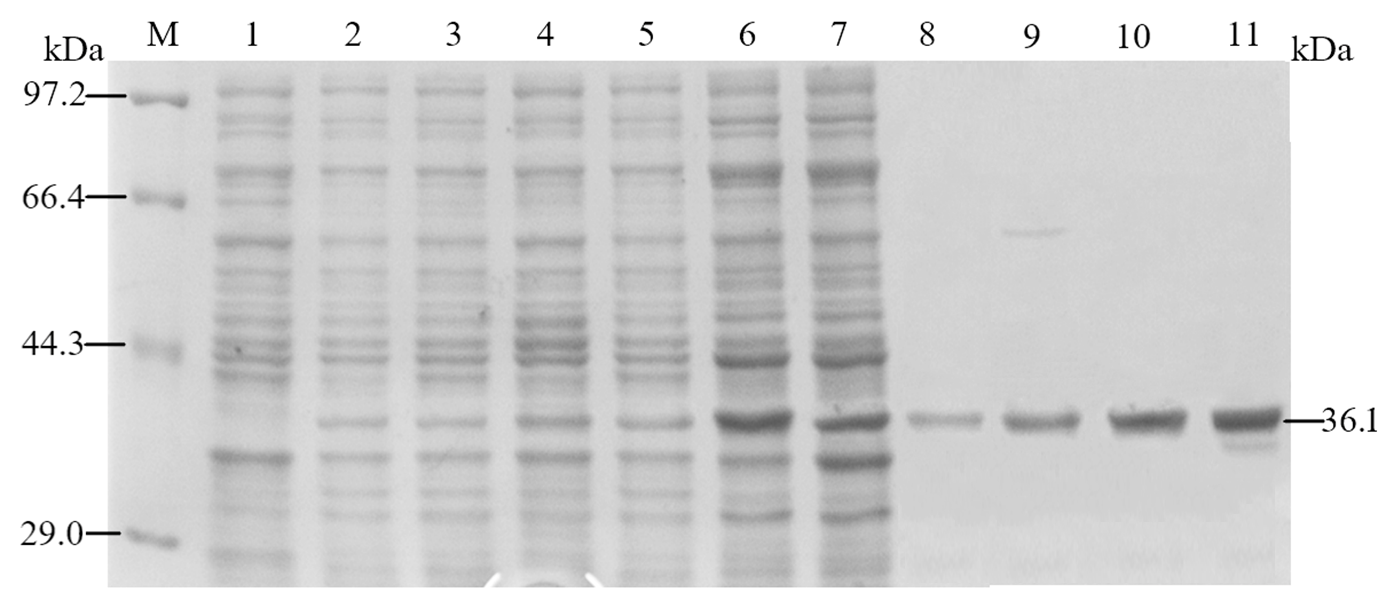


**Supplementary Fig. 7. SDS-PAGE analysis of the expressed and purified EHs.** Lane M, standard proteins; lanes 1–7, the supernatants of *E. coli*/pET-28a, /*pveh3*, /*pveh3*^L128F^, /*pveh3*^L129M^, /*pveh3*^E134K^, /*pveh3*^T137P^ and /*pveh3*^T137P/E134K^ cell lysates; lanes 8–11, the purified *Pv*EH3, *Pv*EH3^E134K^, *Pv*EH3^T137P^ and *Pv*EH3^T137P/E134K^. (Due to the unavoidable problem of the photographic apparatus in our lab, this figure was generated by overexposure)


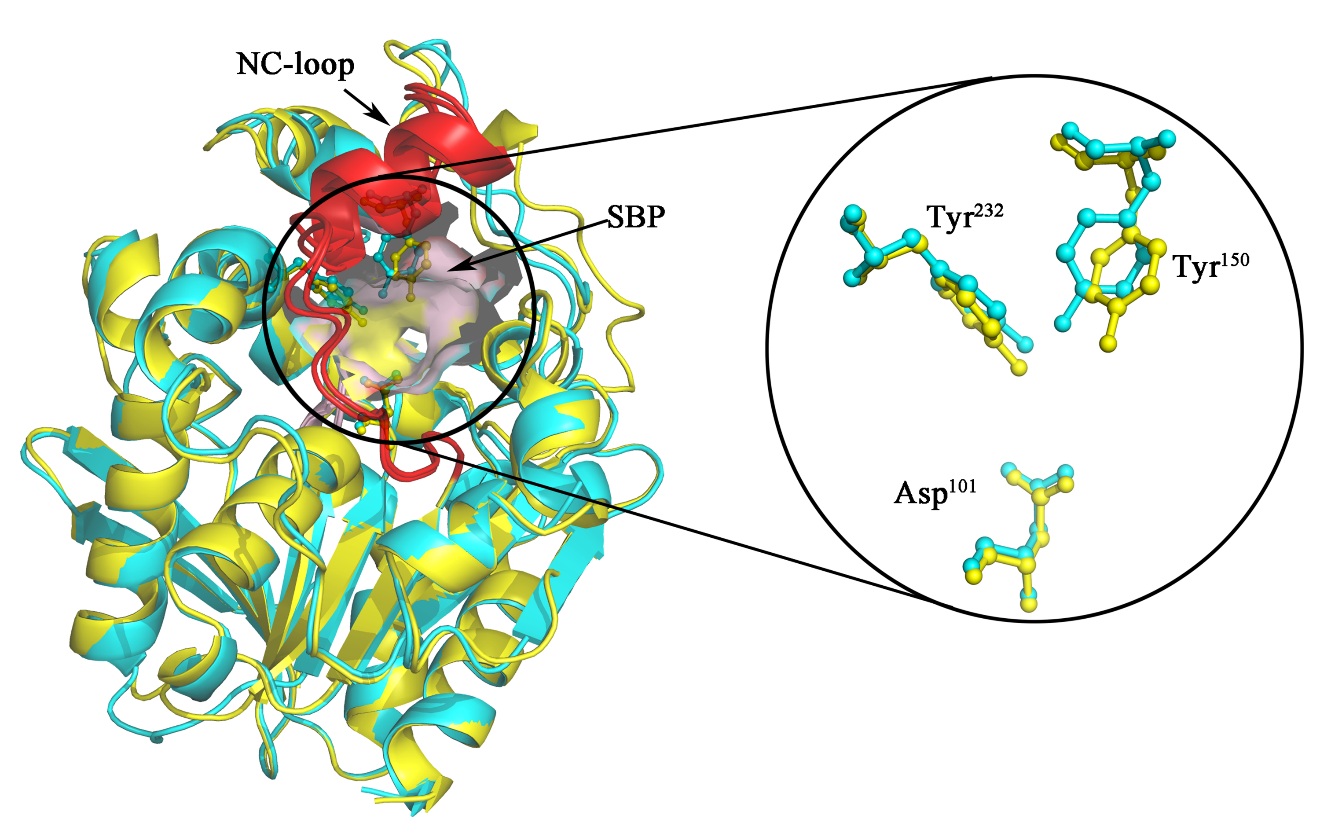


**Supplementary Fig. 8. The 3-D structure alignment between *Pv*EH3 and *Pv*EH3^E134K/T137P^.** The spatial position and orientation of substrate-binding residues, Asp^101^, Tyr^150^ and Tyr^232^, in *Pv*EH3 (or *Pv*EH3^E134K/T137P^) were indicated by cyan (or yellow) sticks.
